# Penetrating Ballistic Trauma to the Popliteal Fossa Without Neurovascular Injury Despite High-Risk Trajectory: An Unusual Case Report

**DOI:** 10.7759/cureus.111483

**Published:** 2026-06-25

**Authors:** Divya Ravikumar, Qurban Babayev, Elchin Orujov, Huseyn Aliyev, Nicat Settarov

**Affiliations:** 1 Department of Trauma and Orthopaedics, Azerbaijan Medical University, Baku, AZE; 2 Department of Traumatology and Orthopaedics, Scientific Research Institute of Traumatology and Orthopedics, Baku, AZE; 3 Department of Traumatology and Orthopaedics, European International Hospital, Baku, AZE

**Keywords:** ballistic trauma, case report, gunshot injury, knee trauma, neurovascular injury, penetrating trauma, popliteal artery, popliteal fossa

## Abstract

Gunshot injuries involving the popliteal fossa are typically considered limb-threatening due to the dense concentration of major vascular and neural structures within the confined anatomical space posterior to the knee joint. Penetrating trauma to this region commonly results in injury to the popliteal artery, popliteal vein, tibial nerve, or common peroneal nerve, often necessitating urgent vascular or reconstructive intervention. Preservation of neurovascular integrity following ballistic trauma to the popliteal fossa is exceptionally uncommon.

We report a rare case of an isolated gunshot injury to the popliteal fossa without associated vascular or neurological damage in a 45-year-old male. Clinical examination at presentation demonstrated intact distal perfusion with symmetric pulses, normal ankle-brachial indices (ABI), and preserved motor and sensory function despite the high-risk trajectory of the projectile. Bedside Doppler ultrasound confirmed patent triphasic distal flow. Given the high risk of deep-tract contamination and mechanical joint compromise, immediate emergency surgical exploration, debridement, and bullet removal were performed through a posterior approach with an on-call vascular surgery team on standby. Postoperative computed tomography angiography (CTA) and magnetic resonance imaging (MRI) confirmed absolute neurovascular, ligamentous, and articular integrity, revealing only an isolated posterior femoral cortical defect.

This case highlights the importance of meticulous neurovascular assessment and prompt surgical management in penetrating injuries around the knee joint. It also demonstrates that ballistic injuries to the popliteal fossa may rarely occur without compromise of adjacent critical structures despite the anatomical vulnerability of the region. While standard trauma algorithms advocate nonoperative imaging surveillance in stable patients without "hard signs" of vascular injury, immediate surgical exploration remains indicated when macro-contamination or mechanical joint threats are present.

## Introduction

The popliteal fossa is a clinically significant anatomical region located posterior to the knee joint and contains essential neurovascular structures, including the popliteal artery, popliteal vein, tibial nerve, and common peroneal nerve [1]. Due to the close proximity of these structures, penetrating injuries involving the popliteal fossa are associated with a high risk of vascular compromise, neurological deficits, limb ischemia, and long-term functional disability [2,3]. Among these injuries, gunshot wounds represent a particularly challenging form of trauma because ballistic trajectories are often unpredictable and capable of causing extensive soft tissue and neurovascular destruction.

Popliteal artery injury is considered one of the most serious complications associated with ballistic trauma around the knee joint, carrying a significant risk of hemorrhage, thrombosis, compartment syndrome, and limb loss if diagnosis or treatment is delayed. Similarly, injuries to the tibial or common peroneal nerves may result in persistent motor and sensory impairment. Consequently, prompt evaluation and management are essential in all patients presenting with penetrating trauma to this region.

Although several reports in the literature describe gunshot injuries to the popliteal fossa with associated vascular or nerve involvement, cases demonstrating complete preservation of neurovascular structures remain exceedingly rare [4]. We present a unique case of a gunshot injury to the left popliteal fossa in a 45-year-old male in whom no vascular or neurological injury was identified clinically, surgically, or radiologically despite the projectile traversing a high-risk anatomical region.

Because of the potential for occult vascular injury and delayed thrombotic complications, penetrating trauma around the knee joint requires meticulous serial neurovascular examination and appropriate imaging evaluation even in patients without overt ischemic findings. The present case is particularly unusual because the projectile traversed a high-risk anatomical corridor while completely sparing the major neurovascular structures of the popliteal fossa.

Neurovascular preservation following a central penetrating ballistic trajectory through the popliteal fossa is exceptionally uncommon due to a combination of dense anatomical geometry and ballistic biomechanics. Anatomically, the popliteal artery, popliteal vein, tibial nerve, and common peroneal nerve are tightly packed within a narrow, diamond-shaped corridor bounded by inelastic fascial planes and rigid musculoskeletal borders [4]. Unlike the thigh or calf, this region lacks thick, intervening muscular bellies to act as a protective buffer. Biomechanically, ballistic injuries damage tissue not only through direct transection but also via acoustic shockwaves and temporary cavitation generated by kinetic energy transfer. Within such a highly confined space, these forces typically induce severe secondary traction or thermal injuries, leading to intimal tearing, acute vascular thrombosis, or irreversible nerve sheath disruption [3]. For a projectile to traverse this dense central pathway without causing functional deficit represents a highly anomalous micro-trajectory.

## Case presentation

A 45-year-old male presented to the emergency department approximately 45 minutes following a gunshot injury involving the left popliteal fossa region. The entrance wound was identified as a clean, low-velocity punctate defect measuring approximately 1 × 1 cm over the lateral aspect of the left knee region, displaying minimal margins of thermal necrosis and no gross structural debris or active external hemorrhage. The patient was hemodynamically stable upon arrival and reported localized pain without active hemorrhage.

Within 15 minutes of arrival, the initial trauma evaluation demonstrated no signs of vascular compromise. Distal pulses, including the dorsalis pedis and posterior tibial arteries, were easily palpable and symmetrical bilaterally. Capillary refill was under two seconds, and the affected extremity remained warm and well perfused. An initial bedside ankle-brachial index (ABI) was performed bilaterally, yielding a normal value of 1.0. To exclude occult vascular trauma before transitioning to advanced imaging, a handheld bedside Doppler ultrasound evaluation was conducted in the emergency department within 30 minutes of admission, confirming normal triphasic flow in both distal arteries. No expanding hematoma, thrill, bruit, active bleeding, or signs of compartment syndrome were observed. Given the high risk of tract contamination and mechanical joint locking, the patient was transitioned to the operating room for emergency surgical exploration exactly 90 minutes following initial presentation.

Neurological examination revealed intact motor and sensory function throughout the left lower extremity. There was no evidence of tibial nerve or common peroneal nerve dysfunction. Active ankle and toe movements were preserved, and no sensory deficits were identified.

Initial plain radiographs of the left knee demonstrated a retained metallic projectile within the popliteal region without evidence of major fracture, joint dislocation, or gross osseous disruption (Figure 1). Given the anatomical proximity of the retained projectile to critical neurovascular structures within the popliteal fossa, and the high suspicion of potential mechanical joint compromise or deep-tract contamination from the projectile, emergency surgical exploration and bullet removal were planned. 

**Figure 1:**
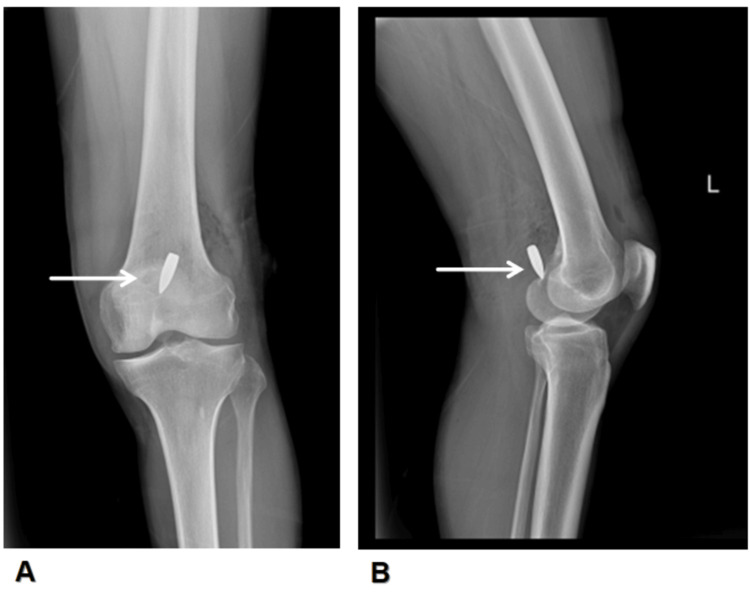
Plain radiograph of the left knee demonstrating retained metallic projectile within the popliteal region without major fracture. (A) Anterior-posterior (AP) view. (B) Lateral view.

In standard trauma algorithms, such as Advanced Trauma Life Support (ATLS) and East Association for the Surgery of Trauma (EAST) guidelines, a hemodynamically stable patient presenting with a proximity extremity injury but no "hard signs" of vascular trauma and a normal ABI (>0.9) is typically managed with noninvasive imaging surveillance or preoperative computed tomography angiography (CTA). However, in this case, the clinical decision pathway was driven by a high suspicion of acute macro-contamination along the deep ballistic tract, introducing an imminent risk of deep-space or joint capsule infection, and the potential for the deeply retained projectile to cause acute mechanical joint locking or secondary soft tissue erosion during early knee movement. Given immediate operating room availability, the surgical team prioritized urgent formal debridement and foreign body extraction over preoperative CTA, relying on direct intraoperative visualization of the neurovascular bundle. An on-call vascular surgery team was formally briefed and remained on active standby throughout the procedure.

A posterior approach to the left lower extremity was utilized to provide direct anatomical access to the popliteal region. Intraoperatively, the projectile was identified adjacent to the posterior cortical surface within the popliteal fossa after careful layer-by-layer soft tissue dissection. Thorough direct neurovascular exploration demonstrated absolute structural preservation of the popliteal artery, popliteal vein, tibial nerve, and surrounding structures. Following closure, a temporary posterior knee splint was applied to immobilize the joint. The rationale for this temporary splinting was to minimize mechanical shear stress on the freshly debrided posterior soft tissues, protect early wound granulation over the complex popliteal skin folds, and prevent sudden involuntary movements that could compromise early soft tissue healing or provoke delayed bleeding. The splint was safely discontinued on postoperative day 5. The wound tract was thoroughly irrigated and debrided following successful bullet extraction (Figure 2). 

**Figure 2:**
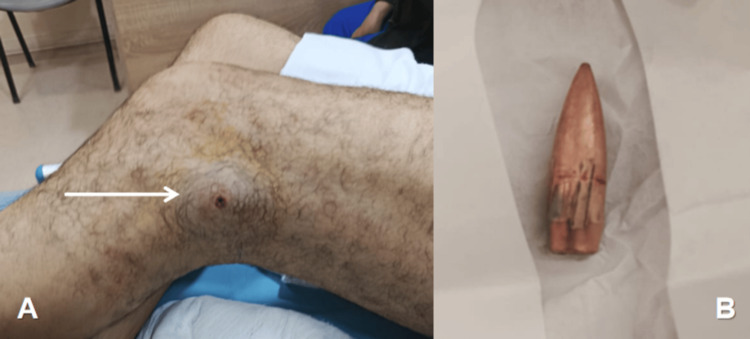
Clinical presentation of the gunshot injury and retrieved projectile. Clinical photograph demonstrating the lateral entry wound over the left knee region at presentation (A) and the bullet retrieved during surgical exploration and extraction of the popliteal fossa (B). The patient exhibited no evidence of vascular compromise or neurological deficit despite the projectile traversing a high-risk anatomical region.

The presumed trajectory of the projectile was from the lateral aspect of the knee toward the posterior popliteal region, ultimately lodging adjacent to the posterior cortical surface while sparing the adjacent neurovascular bundle.

Postoperatively, CT and MRI of the knee joint were performed to further evaluate the extent of osseous and soft tissue injury. CT imaging demonstrated a small focal cortical defect involving the posterior cortex of the distal femur adjacent to the popliteal fossa without comminution or intra-articular extension (Figures 3, 4). MRI confirmed preservation of the adjacent ligamentous, vascular, and neural structures without evidence of hematoma, ligamentous disruption, or occult neurovascular injury (Figure 5). No retained metallic fragments were identified. 

**Figure 3:**
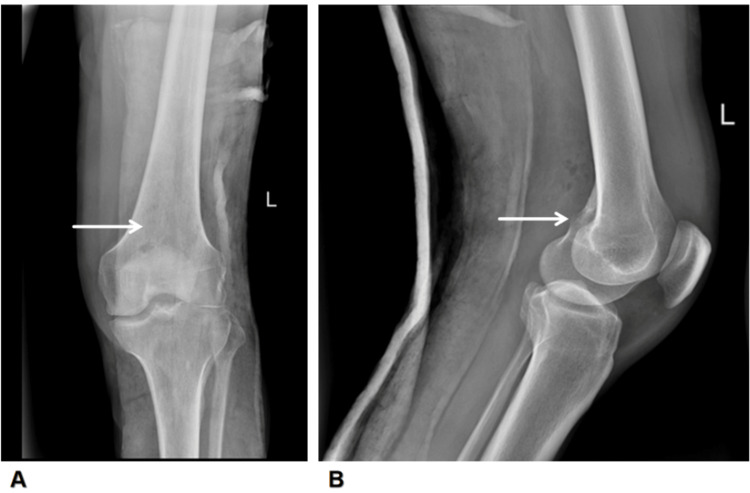
Postoperative radiograph of the left knee demonstrating successful bullet removal and absence of retained metallic fragments. (A) Anterior-posterior view. (B) Lateral view. No residual metallic fragments, acute fracture, or osseous malalignment are identified. A small cortical defect involving the posterior aspect of the distal femur is not clearly appreciable on plain radiography but was subsequently characterized on CT and MRI.

**Figure 4:**
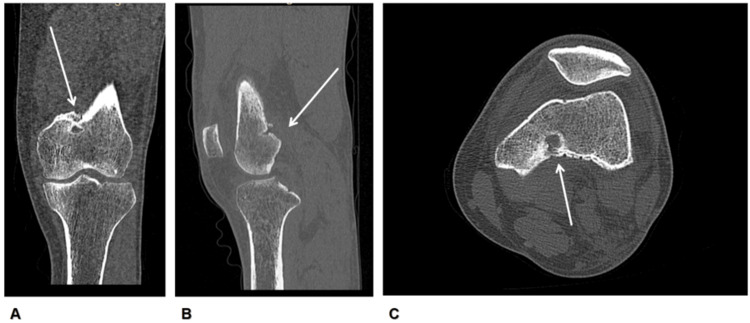
Postoperative CT imaging demonstrating a small focal posterior cortical defect adjacent to the popliteal fossa without intra-articular extension. (A) Anterior-posterior (AP) view. (B) Lateral view. (C) Axial view.

**Figure 5:**
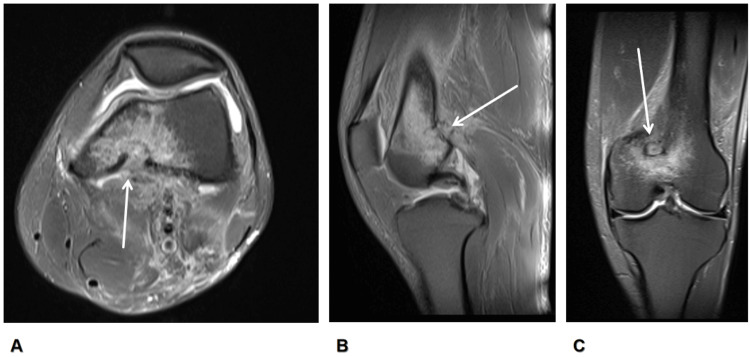
MRI of the left knee confirming preservation of adjacent neurovascular and ligamentous structures without evidence of occult soft tissue injury. (A) Axial view. (B) Lateral view. (C) Anterior-posterior (AP) view.

Following bullet extraction, a temporary immobilization cast was applied for two weeks. Short-term immobilization was utilized to provide soft tissue support, minimize postoperative swelling around the surgical site, protect the wound, and facilitate optimal healing during the early postoperative period (Figures 6, 7). 

**Figure 6:**
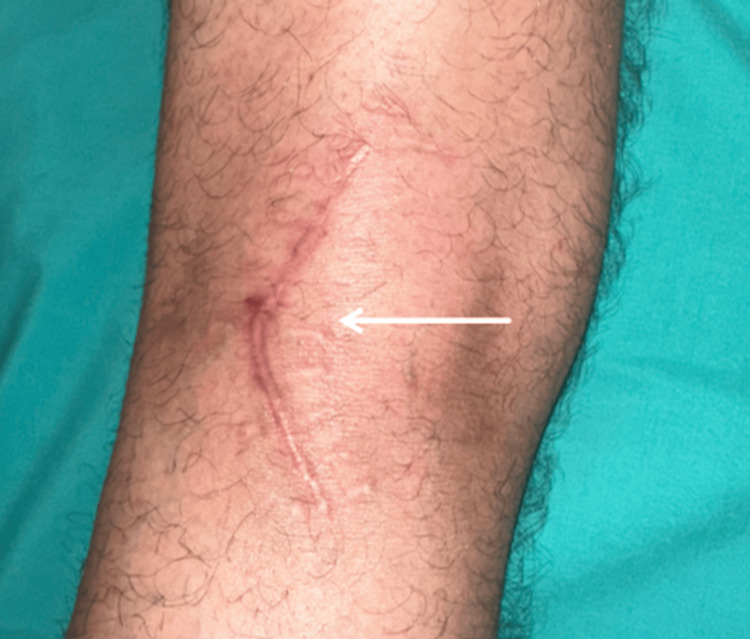
Posterior surgical approach to the popliteal fossa and postoperative scar. Clinical photograph demonstrating the posterior surgical approach utilized for exploration of the popliteal fossa and bullet extraction, and the well-healed postoperative scar at follow-up. The posterior approach provided direct access to the retained projectile while allowing careful assessment of the adjacent neurovascular structures.

**Figure 7:**
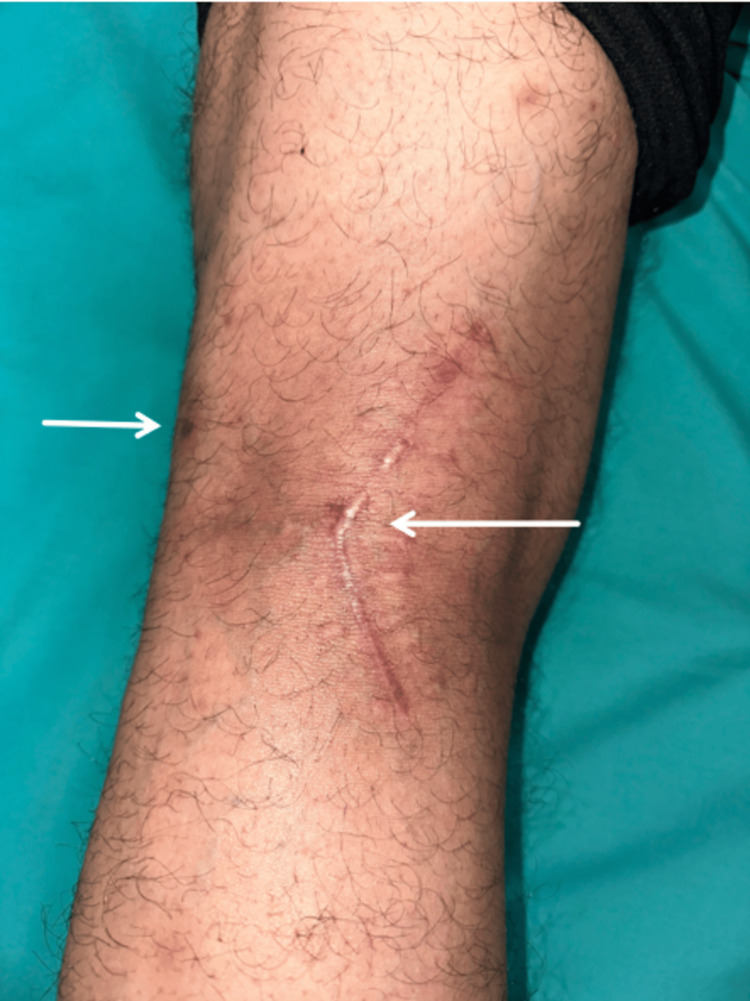
Lateral entry wound and postoperative scar following surgical management of a popliteal fossa gunshot injury. Clinical photographs demonstrating the lateral entry wound over the left knee region at presentation and the healed postoperative scar following surgical exploration and bullet extraction through a posterior approach. The patient maintained intact vascular and neurological function throughout treatment and recovery despite the high-risk trajectory of the projectile.

To definitively rule out occult structural or vascular injury postoperatively, both CTA and MRI of the knee joint were performed. Postoperative CTA was utilized as the primary vascular imaging modality, confirming complete patency of the popliteal artery and vein with no evidence of intimal flaps, pseudoaneurysms, or delayed thrombosis. Postoperative MRI was utilized complementarily to assess the soft tissues and intra-articular structures; it confirmed full preservation of the cruciate ligaments, menisci, and adjacent neural trunks without evidence of deep hematoma or occult nerve sheath disruption.

The postoperative course was uneventful. Serial neurovascular examinations remained normal throughout hospitalization, with preserved distal perfusion and intact motor and sensory function. At the six-week follow-up, the patient demonstrated complete wound healing, preserved knee function, normal ambulation, and no evidence of delayed vascular complications or neurological deficits. At the extended six-month follow-up, the patient demonstrated complete wound healing, excellent knee range of motion, normal unassisted ambulation, completely intact neurological function, and no clinical signs of delayed pseudoaneurysm, arteriovenous fistula, or venous thrombosis.

## Discussion

Gunshot injuries involving the popliteal fossa are generally considered orthopedic and vascular emergencies because of the high concentration of critical neurovascular structures within a relatively confined anatomical space [2,3]. Even low-velocity projectiles may result in devastating consequences depending on trajectory, energy transfer, and tissue cavitation effects. In most reported cases, penetrating injuries to this region are associated with vascular injury, nerve damage, fractures, or combined soft tissue disruption requiring multidisciplinary management [5].

The popliteal artery is particularly susceptible to injury because of its fixed anatomical position adjacent to the femur and posterior knee capsule. Vascular injuries in this region may present with active hemorrhage, absent distal pulses, expanding hematoma, ischemia, pseudoaneurysm formation, or delayed thrombosis [6]. Failure to identify vascular injury promptly can significantly increase the risk of limb loss. Likewise, the tibial and common peroneal nerves are vulnerable to penetrating trauma and may produce severe motor and sensory deficits affecting long-term ambulation and quality of life [6,7].

The present case is noteworthy because the patient sustained a gunshot injury traversing the popliteal fossa without any evidence of vascular or neurological damage. Remarkably, neurovascular integrity was preserved during the initial emergency department evaluation, intraoperative exploration, and postoperative imaging assessment. Such findings are highly unusual considering the anatomical vulnerability of the region.

The absence of neurovascular injury in the present case may be attributable to a tangential ballistic trajectory with limited energy transfer and cavitation effects. Unlike high-velocity ballistic injuries that produce extensive surrounding tissue destruction, low-velocity projectiles may occasionally traverse adjacent to critical anatomical structures without direct disruption [8]. In this patient, the projectile appears to have followed a fortunate anatomical pathway from the lateral knee toward the posterior popliteal region while narrowly avoiding the popliteal artery, popliteal vein, and major neural structures.

This case underscores the critical importance of comprehensive neurovascular examination in all penetrating injuries around the knee joint. The absence of "hard signs" of vascular injury, including active bleeding, absent pulses, bruit, thrill, distal ischemia, or expanding hematoma, may indicate preserved vascular status; however, occult injuries may still exist. Therefore, close clinical observation and appropriate imaging remain essential.

This case fits into an unusual subcategory of trauma management where clinical decision-making deviates from standard nonoperative algorithms. According to current Western trauma algorithms, such as ATLS and EAST guidelines, stable patients with proximity injuries who exhibit normal distal pulses and an ABI >0.9 can be safely monitored or evaluated via preoperative CTA without immediate surgical exploration [9]. This case demonstrates an important caveat to that standard pathway: the absence of "hard signs" of vascular trauma addresses only the hemodynamic status of the limb but does not mitigate mechanical and infectious risks. When a projectile's trajectory introduces macro-contamination into a tightly enclosed fascial space or threatens mechanical architectural locking, immediate surgical exploration can be safely favored over preoperative CTA, provided that baseline perfusion is confirmed via bedside Doppler ultrasound and ABI and a vascular surgery team is available on standby. Advanced postoperative imaging plays an important complementary role in this sequence; while CTA serves as the gold standard to definitively verify vascular integrity, MRI provides essential confirmation that the surrounding nerve trunks and ligamentous complexes suffered no occult blunt kinetic disruption.

Clinical workflow and diagnostic justification

While advanced noninvasive vascular imaging, such as computed tomography angiography (CTA), is frequently performed preoperatively in stable patients presenting with proximity injuries, the clinical decision to proceed directly to surgical exploration in this case was guided by the need for immediate foreign body extraction [9]. This minimized the risk of acute mechanical joint or tendon compromise and addressed potential macro-contamination along the deep ballistic tract within a tightly enclosed compartment. The subsequent postoperative CT and MRI served a highly valuable complementary role in definitively ruling out delayed-onset structural defects, occult intimal flaps, or hidden soft tissue injury.

In the present case, postoperative CT and MRI provided valuable confirmation of the limited extent of injury, demonstrating only a small posterior cortical defect without associated ligamentous, vascular, or neural involvement. Advanced imaging modalities can therefore play an important complementary role in excluding occult structural injury following ballistic trauma [10].

The posterior surgical approach allowed safe access to the retained projectile while enabling direct visualization of the popliteal neurovascular bundle. Early surgical intervention and continuous postoperative neurovascular monitoring contributed to the favorable outcome observed in this patient.

Given the anatomical complexity of the popliteal fossa and the high risk of associated neurovascular injury following penetrating trauma, a broad differential diagnosis was considered during the initial evaluation of this patient. Potential injuries involving the popliteal artery, popliteal vein, tibial nerve, common peroneal nerve, osseous structures, and surrounding soft tissues were systematically assessed through clinical examination, surgical exploration, and postoperative imaging. The differential diagnoses considered and the findings that supported their exclusion in the present case are summarized in Table 1.

**Table 1:** Differential diagnosis of penetrating ballistic trauma to the popliteal fossa and reasons for exclusion in the present case. CT: computed tomography; MRI: magnetic resonance imaging.

Differential Diagnosis	Clinical Features Supporting Diagnosis	Findings Against Diagnosis in Present Case	Relevant Investigations
Popliteal artery injury	Absent distal pulses, active bleeding, expanding hematoma, pallor, cold extremity, delayed capillary refill, limb ischemia	Distal pulses were palpable and symmetrical; no ischemia, hemorrhage, bruit, or expanding hematoma noted during examination or surgery	Vascular examination, Doppler ultrasound, CT angiography, intraoperative exploration
Popliteal vein injury	Significant swelling, venous bleeding, hematoma formation, limb congestion, pain	No venous bleeding, excessive swelling, or hematoma formation observed clinically or intraoperatively	Venous Doppler ultrasound, CT venography, surgical exploration
Tibial nerve injury	Plantar flexion weakness, sensory loss over sole of foot, paresthesia, neuropathic pain	Motor and sensory examination remained completely intact	Neurological examination, electromyography (EMG), MRI
Common peroneal nerve injury	Foot drop, weakness of dorsiflexion, sensory loss over dorsum of foot and lateral leg	No motor deficit, foot drop, or sensory abnormality identified	Neurological examination, nerve conduction studies, MRI
Compartment syndrome	Severe pain out of proportion, tense swelling, paresthesia, pallor, pain with passive movement	No tense compartments, ischemia, or worsening pain observed	Clinical examination, compartment pressure measurement
Popliteal artery pseudoaneurysm	Delayed swelling, pulsatile mass, bruit, recurrent pain, thromboembolic symptoms	No delayed vascular symptoms or pulsatile swelling identified postoperatively	Doppler ultrasound, CT angiography, MRI angiography
Deep soft tissue hematoma	Localized swelling, ecchymosis, tenderness, compressive symptoms	No large swelling or hematoma formation detected clinically or radiologically	Ultrasound, CT scan, MRI
Distal femoral or tibial fracture	Severe pain, deformity, inability to bear weight, crepitus	Imaging revealed only a small posterior cortical defect without major fracture	Plain radiographs, CT scan
Ligamentous injury of the knee joint	Joint instability, swelling, reduced range of motion, mechanical symptoms	MRI showed no ligamentous disruption or intra-articular involvement	MRI of knee joint
Retained foreign body with secondary infection	Persistent pain, swelling, erythema, fever, discharge	Bullet was removed successfully with no evidence of infection	CT scan, MRI, wound assessment, laboratory inflammatory markers
Septic arthritis following penetrating trauma	Fever, severe joint pain, restricted movement, joint effusion	No joint involvement or signs of infection identified clinically or radiologically	Joint aspiration, MRI, inflammatory markers
Occult neurovascular injury	Initially normal examination followed by delayed ischemia or neurological deficits	Serial neurovascular examinations remained normal throughout hospitalization and follow-up	Serial clinical examinations, CT angiography, Doppler studies, MRI

Recent literature consistently emphasizes that penetrating trauma involving the popliteal fossa carries a substantial risk of vascular and neurological injury because of the close anatomical relationship among the popliteal artery, popliteal vein, tibial nerve, and common peroneal nerve [2-4]. Most published reports describe significant vascular compromise, delayed ischemia, pseudoaneurysm formation, thrombosis, or associated nerve deficits following ballistic trauma around the knee joint [10-12].

Research continues to identify popliteal vascular trauma as one of the most limb-threatening extremity injuries, with delayed revascularization, associated fractures, compartment syndrome, and combined vascular injuries remaining major predictors of amputation [11,12]. Contemporary series demonstrate that aggressive limb-salvage strategies incorporating prompt vascular repair, liberal fasciotomy, multidisciplinary orthoplastic management, and early transfer to specialized trauma centers can substantially improve outcomes, even in patients presenting after prolonged ischemic intervals. Gunshot wounds remain a leading mechanism of penetrating popliteal artery injury in modern civilian trauma populations [1-3].

To place the present case within the context of existing evidence, a review of the contemporary literature was performed focusing on popliteal artery and neurovascular injuries associated with gunshot wounds, penetrating trauma, and other traumatic injuries involving the popliteal fossa. Recent studies predominantly report vascular compromise, neurological deficits, limb-threatening ischemia, or the need for complex vascular reconstruction following injuries to this anatomically vulnerable region. In contrast, complete preservation of both vascular and neural structures following a ballistic injury to the popliteal fossa, as observed in our patient, remains exceptionally uncommon. A summary of relevant studies published between 2020 and 2025 is presented in Table 2. 

**Table 2:** Published reports of popliteal vascular and neurovascular injuries following ballistic and penetrating trauma: a review of recent literature.

Year	Authors	Title	Journal	Study Type	Relevance
2025	van Rensburg et al. [1]	Outcomes of Popliteal Artery Injuries in a Level 1 Trauma Centre: A 6-Year Review	European Journal of Trauma and Emergency Surgery	Retrospective review	Includes penetrating injuries; gunshot wounds were the most common mechanism. Evaluates predictors of limb loss and salvage. (Springer)
2024	Tamteki and Ersoy [2]	Our Surgical Results in Popliteal and Infrapopliteal Artery Injuries: 21 Cases without Amputation	Emergency Medicine International	Retrospective series	42.9% of injuries resulted from gunshot wounds; discusses repair techniques and limb salvage. (PMC)
2024	Hussein et al. [3]	Our Experience with Blast and Gunshot Induced Traumatic Vascular Injuries at Somalia’s Major Vascular Referral Center	Scientific Reports	Retrospective study	Large contemporary experience with firearm-related vascular trauma, including lower-extremity vascular injuries. (Nature)
2024	Izawa et al. [4]	Efficacy of Orthoplastic Management in the Treatment of Traumatic Popliteal Artery Injury	Plastic and Reconstructive Surgery Global Open	Clinical study	Discusses multidisciplinary limb-salvage approaches in severe popliteal artery trauma. (PubMed)
2024	Maillard et al. [5]	Femoral Shaft Fracture with Popliteal Artery Injury: A Case Report of a Gunshot Wound	Brazilian Journal of Case Reports	Case report	Directly relevant gunshot-related popliteal artery injury with associated femoral fracture. (bjcasereports.com.br)
2024	Jelínek et al. [6]	Injury of the Popliteal Artery by Shrapnel of a Ukrainian Girl Treated at the University Hospital Ostrava	Casopis Lékařů Českých	Case report	Penetrating popliteal artery injury highlighting atypical presentation despite preserved limb viability. (PubMed)
2024	Zamin et al. [7]	Outcomes of Traumatic Popliteal Artery Injuries at a Level I Trauma Center	Academic Surgical Congress	Institutional review	Contemporary outcomes analysis of traumatic popliteal artery injuries. (Academic Surgical Congress)
2024	Ascoli et al. [8]	The Role of Body Mass Index in High- and Low-Velocity Trauma Causing Knee Injury Associated with Popliteal Artery Lesions	Journal of Orthopaedic Surgery and Research	Retrospective study	Explores outcomes in traumatic popliteal artery lesions associated with knee trauma. (Springer)
2023	Izawa et al. [10]	Traumatic Popliteal Artery Spasm Diagnosed Using Intraoperative Angiography: A Case Report	Trauma Case Reports	Case report	Demonstrates diagnostic challenges in traumatic popliteal artery injuries. (PubMed)
2022	Hundersmarck D et al. [11]	Blunt Popliteal Artery Injury Following Tibiofemoral Trauma: Vessel-First and Bone-First Strategy	European Journal of Trauma and Emergency Surgery	Retrospective study	Important contemporary management paper frequently cited in popliteal vascular trauma literature. (Springer)
2020	Asensio et al. [12]	Popliteal Artery Injuries: Less Ischemic Time May Lead to Improved Outcomes	Injury	Retrospective review	Contemporary limb-salvage and ischemia-time analysis in popliteal artery trauma. (PubMed)

Tamtekin and Ersoy (2024) evaluated 21 patients with popliteal and infrapopliteal arterial injuries and reported gunshot wounds as the most common etiology, accounting for approximately 43% of cases [2]. The authors emphasized that popliteal artery injuries remain among the extremity vascular injuries with the highest risk of amputation and morbidity, highlighting the importance of rapid diagnosis and timely surgical intervention. In their series, the majority of patients required vascular reconstruction procedures, further demonstrating the severe nature of injuries typically associated with this anatomical region.

Similarly, recent case reports continue to describe severe vascular complications following penetrating trauma near the popliteal region. Alcala et al. (2025) reported a gunshot-induced popliteal artery pseudoaneurysm requiring urgent thrombectomy and graft repair despite initially preserved distal pulses [13]. Their findings reinforced that clinically occult vascular injuries may still exist even when overt ischemic signs are absent during the initial evaluation.

Jelínek et al. (2024) described a pediatric shrapnel-related popliteal artery injury without acute limb ischemia, referred to as a "pulseless pink limb," illustrating that vascular injury may present with deceptively mild symptoms. The authors stressed that delayed recognition of arterial compromise may lead to permanent morbidity if not appropriately investigated and treated [6].

Additional contemporary reports further highlight the complexity of ballistic trauma involving the lower extremity. Yatsun et al. (2025) described delayed recognition of femoral artery and vein injury following gunshot trauma, with vascular damage identified several days after the initial event. This case demonstrated that vascular injuries associated with penetrating trauma may occasionally remain clinically occult during early assessment [14].

Although isolated preservation of neurovascular structures following penetrating trauma to the popliteal region is exceedingly uncommon, similar observations have been described in rare nonballistic injuries. Higginbotham et al. (2024) reported a nail-gun injury involving the posterolateral knee in which the common peroneal nerve was pierced intraoperatively without resulting in a neurological deficit [15]. Such reports suggest that, despite the anatomical vulnerability of the region, carefully directed penetrating trajectories may rarely spare critical neurovascular structures.

This case highlights several important clinical considerations in the management of penetrating trauma around the knee joint. First, the absence of "hard signs" of vascular injury does not completely exclude occult arterial damage; therefore, serial neurovascular examinations remain essential. Second, advanced imaging modalities such as CT and MRI may provide valuable assessment of occult osseous, ligamentous, and soft tissue injury even in clinically stable patients. Finally, careful surgical planning based on the anticipated projectile trajectory is critical for safe exploration and foreign body removal in anatomically complex regions such as the popliteal fossa.

Compared with previously reported cases, the present case is unique because the patient sustained a gunshot injury traversing the popliteal fossa without evidence of vascular injury, nerve injury, ischemia, pseudoaneurysm formation, or neurological deficit during emergency evaluation, surgical exploration, postoperative imaging, or follow-up assessment. Imaging demonstrated only a small posterior cortical defect without associated soft tissue destruction or intra-articular involvement. The preservation of neurovascular integrity despite ballistic penetration of this anatomically high-risk region makes this an exceptionally rare clinical presentation and an important contribution to the existing literature on popliteal fossa trauma.

Limitations and Epidemiological Considerations: A primary limitation of this manuscript is its inherent reliance on a single-case report design, which prevents the generalization of clinical outcomes or the formulation of broad trauma management protocols. Furthermore, as is common in civilian emergency medicine, precise forensic ballistic parameters, such as the specific firearm type, projectile caliber, muzzle velocity, firing range, and exact context of delivery, were completely unavailable in the acute medical record.

From an epidemiological perspective, the authors acknowledge that reporting an isolated favorable outcome following penetrating popliteal trauma introduces a distinct risk of survival bias, as patients sustaining catastrophic, immediately fatal, or severely ischemic popliteal injuries are naturally excluded from elective case reporting. Consequently, this paper should not cause clinicians to commit base-rate neglect by underestimating the high statistical probability of devastating neurovascular injury typically associated with ballistic trauma to the popliteal fossa. Instead, this presentation should be interpreted strictly as a micro-trajectory anomaly that emphasizes the clinical value of balancing standard nonoperative surveillance algorithms against immediate patient-specific mechanical and infectious threats.

## Conclusions

Gunshot injuries involving the popliteal fossa are typically associated with significant vascular and neurological complications because of the dense concentration of critical structures within this anatomically confined region. Nevertheless, isolated ballistic injuries without neurovascular compromise may rarely occur despite projectile traversal through a high-risk anatomical pathway. We report a rare case of a 45-year-old male with a gunshot injury to the left popliteal fossa who demonstrated preserved vascular and neurological function during emergency evaluation, surgical exploration, postoperative imaging, and follow-up assessment.

CT and MRI demonstrated only a small posterior cortical defect without associated ligamentous, vascular, neural, or major soft tissue injury. This case emphasizes the importance of meticulous serial neurovascular examination, appropriate imaging evaluation, and carefully planned surgical management in all penetrating injuries involving the popliteal region, even when initial clinical findings appear reassuring.
